# Time-matched accelerometers on limbs and waist in children with CP give new insights into real-life activities after botulinum toxin treatment: A proof of concept study

**DOI:** 10.3233/PRM-210112

**Published:** 2023-04-06

**Authors:** Stefan Gantelius, Sandra Vikerfors, Josefin Jansson Edqvist, Ferdinand von Walden, Maria Hagströmer, Eva Pontén

**Affiliations:** a Department of Women’s and Children’s Health, Karolinska Institutet, Stockholm, Sweden; b Department of Pediatric Orthopaedic Surgery, Astrid Lindgren Children’s Hospital, Karolinska University Hospital, Stockholm, Sweden; c Örebro University Hospital, Örebro, Sweden; d Department of Neurobiology, Care Sciences and Society, Karolinska Institutet, Stockholm, Sweden; e Academic Primary Health Care Centre, Region Stockholm, Stockholm, Sweden

**Keywords:** Accelerometry, adolescent, botulinum toxins, Type A, cerebral palsy, child, extremities

## Abstract

**PURPOSE:**

This study aimed to explore the feasibility of using time-matched uniaxial accelerometers for measuring movement in daily life in children with cerebral palsy (CP) before and after botulinum toxin injections.

**METHODS:**

This observational study of clinical care with a pre-post design was set in the home and school environment. Participants included eleven children (4–13 years of age) with CP (GMFCS I-III). The children wore uniaxial accelerometers (ActiGraph, model GT1M) for 4 days on both wrists, the right ankle and around the waist before, 3 weeks and 3 months after BoNT-A injections in the legs. Five children also got BoNT-A in the most affected arm. All injections were given according to clinical indications and routine. The accelerometers were all time-matched to define ambulation, arm swing, voluntary activity of arms, and bimanual activity. The feasibility of wearing accelerometers with this setup was evaluated. A linear mixed model was used for analysis of the percentage time and at which intensity the different activities were performed. The confidence interval demonstrated any difference between the dominant and non-dominant arm.

**RESULTS:**

Time-matching of accelerometers placed on both wrists, the waist, and one ankle is a feasible method of registering ambulation, arm swing during gait, and arm movements while not ambulating. Before injections, the children spent 5.6% of their time ambulating. This value declined to 3.9% at 3 months. Contrary to clinical goals, arm movement did not increase after injecting the most affected arm with BoNT-A, however, injections may have decreased mirror movements, which are often bothersome for the child.

**CONCLUSION:**

A time-matched 4-accelerometer set-up is feasible in children with cerebral palsy. A future study including time-matched multi-axial accelerometers on all four limbs, could provide important information on the effect of BoNT-A in daily life.

## Abbreviations

CP: Cerebral palsy
BoNT-A: Botulinum toxin type A
GMFCS: Gross Motor Function Classification System

## 1 Introduction

Cerebral palsy (CP) is the most common cause of physical disability and movement disorders in children [1]. The physical disability is strongly influenced by spasticity, which often results in flexed and rotated positions of the elbow, wrist, hip, knee and ankle [2–4].

For more than 20 years, these positions have been attempted to be weakened by injecting botulinum toxin type A (BoNT-A) into the most spastic muscles [5]. The short-term reduction of spasticity is well documented [6–8] and becomes noticeable within days, peaks after approximately three weeks, and typically lasts for a total of 3-6 months [9].

A common treatment goal is to make walking easier with a better arm swing [10]. Another goal is to increase the use of the more affected and spastic arm. Both of these goals are strived for at home and at school. To date, there is no unbiased way of evaluating these clinical treatment effects in daily life of children with CP. Gait, which is mainly an automated movement, can be closely analysed in a motion analysis lab, but does not give information on how, and how much the child actually walks at home. For arm use, there is no gold standard test like gait analysis. Hand and arm movements vary greatly both between and within subjects. They are not automated like gait is, in regard to timing or performance. Treatment goals of improved arm swing and arm use while not ambulating are difficult to assess in a real-life setting. Therefore, a search for a feasible way of testing movements in the home and school environment in children and adolescents with CP is needed. Accelerometers have been employed to quantify both duration and intensity of arm use in adult stroke patients [11]. Four accelerometers placed on both wrists, around the chest and around one ankle have been shown to monitor changes in bimanual activity during stroke rehabilitation [12]. However, distinguishing arm swing during gait from voluntary bimanual activity while sitting, is not clear-cut with this set-up, without time-matching.

Before and after treatment, this study aimed to test if a novel time-matching of ActiGraph raw counts collected from both wrists, the waist and one ankle defining ambulation, arm swing, voluntary arm activity and bimanual activity could be a feasible way of assessing movements in daily life. As common treatment goals are to make walking easier and to increase the use of the more affected arm, the hypothesis was that a change of these parameters would be detected with the new method.

## 2 Material and methods

This is an observational study of standard clinical care with a pre-post design. The setting was patients with CP treated with BoNT-A, 2010 and 2012. No power analysis was possible to make before-hand, as the type of analysis was totally novel.

### 2.1 Participants

Eleven children with CP (GMFCS I-III) (5 girls, 6 boys, age 4–13 years) scheduled to receive BoNT-A injections in the legs were included consecutively, first 2010, then 2012. Exclusion criteria were BoNT-A injections or orthopaedic surgery within the previous six months. One child had done a crude pre-injection accelerometer session but was excluded by their own choice. Ethical approval was obtained from the local institutional review board, conforming to the Helsinki Declaration.

All children received BoNT-A injections in one or both legs and 5 were also injected in the more affected arm. In the 2010 cohort, a 3-month follow up was not planned, and therefore not performed (#9, #10, #11). Two in the 2012 cohort were not present at the 3-month follow up (#3, #4) (Table 1).

**Table 1:** Characteristics of subjects with CP, muscles

Subject	Age	CP subtype	Pre-injection Accelerometer setup	Muscles injected	3-week follow-up			3-month follow-up		
					Accelerometers	Occupational therapist	Physio-therapist	Accelerometers	Occupational therapist	Physio-therapist
#1	5y 5m	Bilateral	W, F, DH, NDH	Gastrocnemius bilaterally	W, F, DH, NDH	AHA	Clinical goals	W, F, DH, NDH	Clinical goals	ROM bilaterally
	Boy			Hamstrings bilaterally		Zancolli sin				MAS bilaterally
				Pronator teres sin		House				SMC bilaterally
			Pronator quadratus sin			ROM sin, MAS sin				Clinical goals
#2	4y 9m	Unilateral	W, F, DH, NDH	Gastrocnemius sin	W, F, DH, NDH	AHA	GMFM	W, F, DH, NDH	Clinical goals	ROM bilaterally
	Girl			Tibialis posterior sin			ROM bilaterally			MAS sin
				Adductor pollicis sin			Clinical goals
				Flexor pollicis brevis sin
				Brachioradialis sin
				Brachialis sin
#3	4y 8m	Bilateral	W, F, DH, NDH	Gastrocnemius bilaterally	W, F, DH, NDH	-	ROM bilaterally	-	-	ROM bilaterally
	Boy			Gracilis bilaterally			MAS bilaterally			MAS bilaterally
							Clinical goals			Clinical goals
#4	5y 1m	Bilateral	W, F, DH, NDH	Gastrocnemius bilaterally	W, DH, NDH	-	-	-	-	ROM bilaterally
	Boy			Hamstrings bilaterally						MAS bilaterally
				Gracilis bilaterally						SMC bilaterally
										Clinical goals
#5	13y 6m	Unilateral	W, F, DH, NDH	Hamstrings dx	W, F, DH, NDH	-	-	W, F, DH, NDH	-	ROM bilaterally
	Boy									MAS dx
										SMC dx
										Clinical goals
#6	7y 8m	Bilateral	W, F, DH	Gastrocnemius bilaterally	W, F, DH, NDH	ROM sin	-	W, F, DH, NDH	ROM sin	ROM bilaterally
	Girl			Gracilis bilaterally					Clinical goals	MAS bilaterally
				Adductor longus bilaterally						Clinical goals
				Pronator teres sin
				Pronator quadratus sin
#7	7y 0m	Unilateral	W, F, DH, NDH	Gastrocnemius dx	W, F, DH, NDH	SHUEE	ROM dx	W, F, DH, NDH	SHUEE	ROM dx, MAS dx
	Girl			Tibialis posterior dx		CHEQ	MAS dx		House, Zancolli	Clinical goals
				Extensor hallucis dx		House, Zancolli	Clinical goals		ROM dx, MAS dx
				Brachialis dx		ROM dx, MAS dx
				Brachioradialis dx
				Pronator teres dx
				Adductor pollicis dx
#8	10y 2m	Bilateral	W, F, DH, NDH	Gastrocnemius dx	W, DH, NDH	-	-	W, F, DH, NDH	-	-
	Girl			Soleus dx
#9	12y 6m	Bilateral	W, F, DH, NDH	Gastrocnemius bilaterally	W, F, DH, NDH	-	-	-	-	ROM bilaterally
	Girl
#10	10y 11m	Bilateral	W, F, DH, NDH	Gastrocnemius dx	W, F, DH, NDH	-	-	-	-	ROM bilaterally
	Boy			Soleus dx
#11	5y 4m	Bilateral	W, F, DH, NDH	Gastrocnemius bilaterally	W, F, DH, NDH	-	-	-	MUUL	ROM bilaterally
	Boy			Biceps brachii dx					House dx, Zancolli dx	MAS bilaterally
									ROM dx, MAS dx

CP=Cerebral Palsy, Accelerometer setup: *W* = waist, *F* = foot, DH = dominant hand, NDH = non dominant hand. ROM = Range of Motion, MAS = Spasticity according to the Modified Ashworth Scale, AHA = Assisting Hand Assessment, SMC = Selective Motor Control, CPUP = Follow-up according to the Swedish National CP Registry, GMFM = Gross Motor Function Measure, SHUEE = Shriners Hospital Upper Extremity Evaluation, CHEQ = Children’s Hand-use Experience Questionnaire, House = House functional classification system, Zancolli = Zancolli Spastic Hand Evaluation, MUUL = the Melbourne Assessment of Unilateral Upper Limb Function, Sin = left, Dx = right, ”-”=not performed, *y* = years, *m* = months.

### 2.2 Clinical assessments

Physiotherapists and occupational therapists performed clinical assessments before, three weeks and three months post-injections. Treatment goals were set together with the child and parents, in some cases, aided by standardized video assessments.

All children included in the study received home-based goal-oriented post injection training. Habilitation physiotherapists and occupational therapists met the child after the injections during which the family got training instructions based on pre-defined individual goals of the treatment.

Clinical follow-up records from both the hospital and the habilitation centres were reviewed (Table 1) for information of the feasibility of the accelerometer use.

### 2.3 Injections with BoNT-A

Based on body weight, physical examination, and treatment goals, the orthopaedic surgeon decided on the dose of BoNT-A (Botox^®^, Allergan, Irvine, CA, USA) and which muscles to inject [5].

### 2.4 Accelerometer set-up and analysis

The ActiGraph GT1M is a uniaxial accelerometer with the dimensions 3.8x3.7x1.8 cm, approximately the size of a wrist watch, detecting vertical accelerations in the magnitude from 0.05 to 2.00 G, equipped with a filter discriminating human movements from vibrations. The output from the monitors was sampled 10 times per second and summed over a selected time interval called epoch, which was set to one second. The total of accelerations was transformed into ActiGraph ‘counts’. Accelerometers were attached to both wrists, the waist and around the right ankle using soft elastic bands. They were worn for four consecutive days, including two weekdays and an entire weekend during all waking hours, but were not worn during activities involving water, i.e. showering or swimming [13]. In a diary, parents recorded any unusual physical activity or inactivity, such as bed rest due to illness, which was taken into account regarding the feasibility of the method. Except for the one occasion with a torn elastic band, patients and families did not report any problems or inconvenience from wearing the accelerometers.

Since walking and running require reciprocal movement by both legs, ambulation was monitored by combining data from the waist accelerometer with one on one ankle. With accelerometers on both wrists, the dominant and the non-dominant sides were compared. Repeated longitudinal assessments of actigraphy parameters were collected with the intention to describe a) time spent ambulating, b) acceleration during ambulation, c) symmetry of arm swing, d) voluntary activity of the arms and e) bimanual activity, as part of voluntary activity.

### 2.5 Missing data

Subject 4 had a torn elastic band on the foot accelerometer at 3 weeks, and no data was recorded. For subject 8, the foot accelerometer at 3 weeks did not load data and, for subject 6, the accelerometer on the pre-injection non-dominant arm did not load data.

Subjects 3, 4, 9, 10 and 11 had no 3-month accelerometer follow-up (Table 1).

### 2.6 Data collection and analysis

A prospective collection of accelerometer data and evaluation of feasibility was done. The accelerometers were programmed with the ActiLife Data Analysis software (ActiLife 6 v9.0) and the epoch time was set to one second. The data were obtained independently from accelerometers on the dominant (dom) and non-dominant arms (ndom), waist (W) and leg (L) [11] and were synchronized with respect to time (t).

A customized program for data processing was developed in MatLab^®^ 7.9.0 (R2009b; Matrix Laboratory, MathWorks, Natick, MA, USA). For this purpose, counts per second were first adjusted to active time by subtracting non-wear time (periods during which the values recorded by all of the accelerometers were zero for 5 consecutive minutes) from the total recording time. The data for each day were analysed independently, dividing active time into time spent performing ‘voluntary activity’ or ‘ambulation’ (walking, running, jumping), expressed as % time/time wearing the accelerometers. Voluntary activity was defined as acceleration of the waist of < 25 counts/second (cps) along with a detectable acceleration of one or both arms. Together with a detectable acceleration of the ankle, ambulation was defined as a recorded acceleration of the waist of > 25 cps, previously measured during slow walk in children with CP and acquired brain injury [14, 15].

Arm swing was defined as arm movements (cps) during ambulation. The degree of arm swing symmetry was estimated as the cps difference between the arms, subtracting non-dominant cps from the dominant arm cps.

Bimanual activity was defined as both arms having an acceleration of > 0 cps and the waist an acceleration of < 25 cps. It was expressed as a percentage of the time spent performing a voluntary activity (Table 2). The intensity of an activity was presented as the mean counts per second, as a measure of mean acceleration.

**Table 2:** Definition of activities and terms

Activity	Definition
Ambulation	W>25 cps+L>0 cps
**t_a_**	time spent ambulating
Ambulation, %	t_a_/active time
**During ambulation:**
Acceleration of the leg, counts/sec	sum(L)/t_a_
Acceleration of the dominant arm, counts/sec	sum(dom)/t_a_
Acceleration of the non-dominant arm, counts/sec	sum(ndom)/t_a_
Acceleration of the waist, counts/sec	sum(W)/t_a_
**Voluntary activity of arms=**	(*W* < 25 cps+arm_dom_>0 cps and/or
**Using one or both arms while sitting or lying down**	*W* < 25 cps+arm_ndom_>0 cps)
**t_*v*_**	time spent in voluntary activity
**Voluntary activity of arms, % **	t_v_/active time
**During voluntary activity:**
Time spent using the dominant arm (%)	t_dom_/t_v_
Time spent using the non-dominant arm (%)	t_ndom_/t_v_
Acceleration of the dominant arm counts/sec	sum(_dom_)/t_v_
Acceleration of the non-dominant arm counts/sec	sum(_ndom_)/t_v_
**Bimanual activity**, i.e., time spent using the arms simultaneously while sitting or lying down	t_sim_/t_v_
**During bimanual activity**
Acceleration of the dominant arm counts/sec	sum(_dom_)/(t_sim_/t_v_)
Acceleration of the non-dominant arm counts/sec	sum(_ndom_)/(t_sim_/t_v_)

W=waist worn accelerometer, *L* = leg worn accelerometer, cps = counts/second, t_a_ = time spent in ambulatory activity, active time = time accelerometers were worn, sum = the sum of acceleration (counts), t_dom_ = total time when acceleration of the dominant arm was > 0, t_ndom_ = total time when acceleration of the non-dominant arm was > 0, t_v_ = time spent by the arms performing voluntary activity, t_sim_ = time when acceleration of both arms was > 0.

### 2.7 Statistical analysis

The statistical analyses were performed with the IBM SPSS Statistics 24 software (IBM, NY, US). A linear mixed model was used to analyse outcomes in order to account for repeated measures, within-subject variance, correlated data and missing values; see Appendix for more details. An interaction term was introduced into the model to examine for heterogeneity. Regarding differences between the arms, values (acceleration or % of the time), were subtracted Dominant –Non-dominant. If the confidence interval did not include 0, the difference between the arms was significant.

## 3 Results

### 3.1 Time spent ambulating as percentage of wear-time

For the entire group, the mean percentage of time spent ambulating was 5.6% prior to the botulinum toxin injections in the legs. Three weeks after the injections, the time ambulating was reduced to 4.7 % (*p* = 0.049), and the decline persisted after three months (3.9%) (*p* = 0.022) (Tables 3 and 4). Children who were injected with BoNT-A in the leg(s) together with the non-dominant arm spent the same percentage of time ambulating, across all time points, as those treated in the legs only (Table 5).

**Table 3:** Activity time % and acceleration before and after BoNT-A injections

	Pre-injection		Three weeks post-injection		Three months post-injection	
	Mean	95% CI	Mean	95% CI	Mean	95% CI
**Ambulation, whole group**	5.6 %	3.1; 8.0	4.7 %	2.3; 7.1	3.9 %	1.4; 6.5
% of time when accelerometers were worn
**Ambulation, whole group**	69.7	58.0; 81.2	70.3	58.7; 81.9	71.5	59.5; 83.5
Acceleration of the waist (cps)
**Voluntary activity of the arms, whole group**	41.6	31.7; 51.5	36.3	26.4; 46.2	40.6	25.4; 55.8
% of time when accelerometers were worn
Dom arm use as % time in voluntary activity	79.7	73.6; 85.8	83.7	77.6; 89.7	89.8	82.2; 97.4
Group **with** BoNT-A injection into one arm	52.7	37.0; 68.3	40.7	25.0; 56.3	46.0	30.3; 61.7
Group **no** BoNT-A injection into one arm	30.6	18.5; 42.7	32.0	19.9; 44.1	35.3	9.0; 61.5
*Diff dom-ndom % time* in voluntary activity Group **with** BoNT-A injection into one arm	17.0	**5.0; 29.0**	23.7	**11.7; 35.6**	22.3	**10.4; 34.3**
*Diff dom-ndom % * time in voluntary activity Group **no** BoNT-A injection into arm	-1.7	-9.5; 6.1	-0.4	-8.3; 7.4	9.8	-2.5; 22.1
**Voluntary activity of the arms, whole group**
Acceleration *difference, dom-ndom (cps)*	8.3	-10.7; 27.3	22.6	**3.7; 41.7**	21.5	**0.8; 42.1**
Group **with** BoNT-A injection into one arm	23.4	-8.4; 55.2	43.4	**11.6; 75.2**	36.3	**4.5; 68.1**
Group **no** BoNT-A injection into one arm	-6.8	-27.6; 14.7	2.0	-18.8; 22.8	6.7	-20.3; 33.6
**Bimanual activity, whole group**
% of time during voluntary activity	46.0 %	39.3; 52.7	55.7 %	49.0; 62.4	43.9 %	35.0; 52.8
Group **with** BoNT-A injection into one arm	54.7 %	43.5; 65.9	55.0 %	43.8; 66.2	60.0 %	48.8; 71.2
Group **no** BoNT-A injection into one arm	37.3 %	30.0; 44.6	56.4 %	49.1; 63.8	27.8 %	14.1; 41.5
**Bimanual activity, whole group**
Acceleration *difference, dom-ndom (cps)*	4.3	-16.7; 25.2	24.2	**3.2; 45.1**	22.3	-1.1; 45.7
Group **with** BoNT-A injection into one arm	15.5	-19.5; 50.6	45.3	**10.2; 80.4**	38.8	**3.7; 73.8**
Group **no** BoNT-A injection into one arm	-7.0	-29.9; 16.0	3.0	-19.9; 26.0	5.8	-25.8; 37.6

cps=counts/second, dom = dominant arm, ndom = non-dominant arm, CI = Confidence Interval. When *(dominant - non-dominant*) has a confidence interval not including 0, the difference between the arms is significant on a 5% level, and is marked in bold.

**Table 4:** Comparisons pre- and 3 weeks and 3 months post-injections

	Mean	SEM	95% CI	*p*-value
	difference
**Ambulation, whole group**
% of total time
Pre v/s 3 weeks	0.9	0.40	0.0; 1.8	**0.049**
Pre v/s 3 months	1.6	0.63	0.3; 3.0	**0.022**
3 weeks v/s 3 months	0.8	0.63	-0.6; 2,1	0.25
**Voluntary activity, whole group**
% of total time
Pre v/s 3 weeks	5.3	5.7	-8.2; 18.8	0.386
Pre v/s 3 months	1.0	7.9	-16.7; 18.7	0.902
3 weeks v/s 3 months	-4.3	7.9	-22.0; 13.4	0.598
**Voluntary activity**
Acceleration *difference*
*dom-ndom arm (cps)*
Group leg(s) **with**
injection of BoNT-A into one arm
Pre v/s 3 weeks	20.0	10.1	-41.5; 1.5	0.066
Pre v/s 3 months	-12.9	10.7	-34.4; 8.6	0.22
3 weeks v/s 3 months	7.1	10.7	-14.4; 28.6	0.49
**Bimanual activity**
% of time spent performing voluntary activity
Group leg(s) **no** injection of BoNT-A into one arm
Pre v/s 3 weeks	-19.1	4.99	8.5; 29.8	**0.002**
Pre v/s 3 months	9.5	7.52	-6.1; 25.1	0.22
3 weeks v/s 3 months	28.6	7.52	13.0; 44.2	**0.001**
**Bimanual activity**
Acceleration *difference*
*dom-ndom arm (cps)*
Group leg(s) **with** injection
of BoNT-A into one arm
Pre v/s 3 weeks	-29.8	12.7	-57.0; -2.6	**0.03**
Pre v/s 3 months	-23.2	12.7	-50.4; 4.0	0.09
3 weeks v/s 3 months	6.5	12.7	-20.6; 33.7	0.6

SEM=Standard Error of the Mean, CI = Confidence Interval, cps = counts/second, dom = dominant arm, ndom = non-dominant arm. Differences on a significance level of *p* < 0.05 are marked in bold.

**Table 5:** Comparison of children who got BoNT-A injections in arm and leg(s) with those who got injections only in the leg(s), across all time points

	BoNT-A injection in arm+leg(s)		BoNT-A injection only leg(s)	
	Mean	95% CI	Mean	95% CI	Difference arm + leg(s) vs leg(s) only	95% CI	*p*-value
**Ambulation**	4.4	0.8; 8.0	5.1	1.8; 8,3	0.7	-4.1; 5.5	0.754
% of total time
**Ambulation**	69.0	51.8; 86.0	72.1	56.7; 87.4	3.1	-19.8; 26.1	0.764
Acceleration waist (cps)
**Ambulation**
Acceleration arms
*Difference dom-ndom (cps)*	27.3	-1.9; 56.5	-10.9	-35.2; 13.5	38.1	0.4; 75.8	**0.048**
**Voluntary activity of the arms**	46.4	32.1; 60.8	32.6	20.7; 44.5	13.8	-4.0; 31.7	0.107
% of total time
**Voluntary activity of the arms**
% of total time	21.0	**10.2; 31.8**	2.5	-4.9; 10.0	18.4	5.4; 31.5	**0.011**
*Difference dom-ndom % points*
**Voluntary activity of the arms**
Acceleration	34.3	**3.9; 64.8**	0.6	-19.8; 21.1	33.7	-3.0; 70.3	0.68
*Difference dom-ndom (cps)*
**Bimanual activity**
% of time in voluntary activity	56.6	48.9; 64.2	40.5	34.5; 46.5	16.1	6.4; 25.7	**0.004**
**Bimanual activity**
Acceleration
*Difference dom-ndom (cps)*	33.2	**0.2; 66.2**	0.7	-21.8; 23.0	32.5	-7.3; 72.4	0.09

BoNT-A=botulinum toxin type A, cps = counts/second, CI = Confidence Interval, dom = dominant arm, ndom = non-dominant arm. Significant differences between treatment groups, *p* < 0.05, are marked in bold. When (dominant - non-dominant) has a confidence interval not including 0, the difference between the arms is significant on a 5% level, here marked in bold.

### 3.2 Acceleration during ambulation

For the whole group, acceleration of the waist during ambulation was the same before and after the BoNT-A injections (Table 3), with no difference between children who got BoNT-A in the legs only and those injected in one or two legs plus one arm (Table 5).

### 3.3 Symmetry of arm swing

At neither time point there was significant acceleration difference between arms irrespectively of whether the children got BoNT-A in the arm or not. However, across all time points, children who received BoNT-A in the arm had a larger acceleration difference between arms compared to those who did not (*p* = 0.048) (Table 5).

### 3.4 Voluntary activity of the arms

The time spent performing voluntary activity, i.e. moving one or both hands while sitting or lying down, did not change looking at the whole group across all time points. There was no relationship (interaction) with getting BoNT-A injections in the most affected arm or not (Tables 3, 4).

With the aim to improve arm function, children who got BoNT-A in the arm used their hands voluntarily 53% of the time before injections and, contrary to clinical goals, did not increase that time after injections, using their hands 41% of the time at three weeks and 46% at three months post injections (Table 3). Across all timepoints, these children also had a significant difference between the arms concerning the time they were active, which was not the case for the children who got BoNT-A in the leg(s) only (*p* = 0.011). The dominant arm was used 21 percentage points more than the non-dominant arm, while for the children injected in the legs only, the difference was only 2.5 percentage points regarding time (Table 5).

The clinical goal was to increase the usage of the non-dominant arm. On the contrary, a small non-significant increase in the voluntary use of the non-injected dominant arm at the 3-week and 3-month time points and a small, also non-significant, decrease in the use of the injected arm (data not shown) was observed.

Concerning acceleration difference between arms, contrary to the hypothesis, there was a trend that this difference increased at 3 weeks (*p* = 0.066) (Tables 3 and 4).

### 3.5 Bimanual activity, as part of voluntary activity

In general, comparing the groups across all time points, voluntary activity was more bimanual in children with indications for BoNT-A in the legs plus the most affected arm (*p* = 0.004) (Table 5). Before injections, 55% of their voluntary arm use was bimanual, compared to 37% for the children who got BoNT-A only in the legs (*p* = 0.013) (Table 3), but did not change after injections. This contrasts to the group that only got injections in the legs; bimanual activity increased to 56% of voluntary activity 3 weeks post injections (*p* = 0.002), and then decreased again 3 months after injections to 28% (*p* = 0.001) (Table 4).

No difference in acceleration between the arms in the children who had indications for BoNT-A in the arm and leg(s) before the injections was detected. However, three weeks after the injections, the acceleration difference between the arms was significantly different (*p* = 0.034) from before injections due to both increased acceleration in the dominant arm, and decreased acceleration in the injected non-dominant arm (Tables 3 and 4). After 3 months, the difference between the arms was still significant (Table 3).

## 4 Discussion

Here, it is demonstrated how a totally new way of time-matching four ActiGraph GT1M accelerometers during four days in a real-life environment is a feasible way of providing information on ambulation and arm movements in children with CP, which is not obtained through standard follow up of BoNT-A treatment. These accelerometers have been widely used to monitor general physical activity by attaching the accelerometer to the waist. Upper limb function, such as bimanual movement after constraint induced movement therapy CIMT has been evaluated [16]. However, arm swing during gait is then also included in the analysis. By combining accelerometers on both wrists, the waist and one ankle new information on movements in daily life has been obtained. Due to a misunderstanding and problems with the strapping, two subjects did not wear the ankle accelerometer pre-injection, nor at the three-week follow-up. For another subject, the accelerometer on the non-dominant arm was not functioning pre-injection. Otherwise, the accelerometer monitoring was satisfactory. With a future larger study, it would be preferable to use state-of-the art multiaxial accelerometers and also include both ankles instead of only one. With time-matching, different movement patterns occurring in daily life can then be accurately collected while the subject is not observed. Machine learning techniques use so-called training data to make predictions and may further improve analyses of this complex data. In order to help set the hypotheses for the next study, the data obtained in this feasibility study is analysed and discussed.

First, contrary to expectations, the total time the children ambulated decreased after BoNT-A treatment. Second, BoNT-A treatment of the more impaired hand and arm did not increase the voluntary use of that hand, which was one of the clinical goals. Instead, the data might be interpreted as a decrease in mirror movements, which are involuntary simultaneous and similar movement of the opposite hand when performing a task with one hand. Mirror movements are common and often bothersome for children with CP.

### 4.1 Ambulation

When planning the BoNT-A treatments, the aim was to improve the muscle balance around the joints by injecting muscles that were relatively over-active, e.g., the gastrocnemius in children who walked on their toes and the hamstrings in those who had a crouch gait [17]. While some 3D gait analysis follow-up studies have verified the intended effect of the injections, with a larger dorsiflexion of the foot and less crouch gait, general gait improvements in daily life after botulinum toxin injections have not been easy to demonstrate [18, 19]. In the current study, the children were ambulatory only 6% of the total time during the day prior to injections. Contrary to expectations, the children ambulated progressively less after BoNT-A treatment, first 5% after 3 weeks and then 4% three months post-injection, i.e. a 30% reduction in ambulatory activity that was already initially quite low. Such a decline in ambulation in this patient group, who were already non-ambulating 94% of all waking hours, is negative in general. As a comparison, ActiGraph measurements of typically developed school children have shown that they are sedentary (<2 cps) 75% of the time they wore the accelerometer around the waist, and in light activity (2-38 cps) in 17% of wear time [20].

BoNT-A not only denervates extrafusal fibres, weakening the injected muscle, but also affects intrafusal fibres, which influence sensory afferents to the spinal cord, the brainstem and cortex. Thus, the spasticity is reduced and feedback changes in the spinal and cortical circuitry can affect muscles distant to the ones initially injected [21]. Axonal transport of BoNT-A is also possible, changing synaptic transmission in both the ventral and dorsal horns in the spinal cord, possibly hampering co-contractions and the typical spastic gait [22]. However, no improvements were seen with respect to gait. A tentative explanation for these findings could be that the early onset peripheral weakness effect made the children feel unsecure so that they preferred to sit and play. In addition, other long-term effects were not caught in this short-term study [23, 24].

### 4.2 Voluntary activity of the arms

The aim of the BoNT-A treatment of the arm was to make it more useful for dual arm tasks (e.g., improving supination and increasing extension of elbow, wrist and thumb). As expected, the injected arm was used less before treatment, but contrary to expectations, the BoNT-A treatment did not increase the use of the injected arm in daily life.

The treatment effect of Hand-Arm Bimanual Intensive Therapy (HABIT) has been tested with accelerometers on each wrist while the children were performing a standardized video-recorded bimanual test, the Assisting Hand Assessment (AHA). Raw accelerometer scores were time-matched to the different tasks in the AHA-film, and the percentage time each arm was used for each task was registered. Treatment with HABIT increased the percentage of the time the more involved arm was moved during AHA tasks from 63% to 78%. Movement of the non-involved arm remained the same (90–94%). The change in amount of use did not correlate to AHA scores, indicating that amount of use is not correlated to quality of use [25]. Also, Beani et al. have also recently shown by accelerometers on each wrist during AHA that children with CP moved their affected arm much less, and that the asymmetry index was larger with higher MACS values [26].

Muscles were selected for injection by observing the child in standardized and bimanual activities. In some studies, injecting relatively over-active muscles has shown to improve hand function [27, 28]. However, a systematic review of randomized controlled studies has not shown evidence of a significant positive effect of BoNT-A injections on manual function [29]. Although, a Cochrane review on BoNT-A as an adjunct to occupational therapy has shown beneficial effects [6].

Surprisingly, the difference between the arms increased after injections. Although injections made it easier to reach and open the hand to grasp, it is plausible that weakness that the child was not accustomed to could have made them reluctant to use that hand in daily life. Another explanation could be that mirror movements in the injected hand decreased. In cerebral palsy, mirror movements are common as the early brain lesion may lead to a structural reorganization of the corticospinal tract [30, 31], leading to an ipsilateral rather than contralateral control of the most affected hand. Manual ability may become somewhat maintained, but often with intense concomitant mirror movements [32–34]. In addition, hypothetically, the increased use of the non-injected arm could be due to non-direct effects on the muscle per se, for example by retrograde neural transportation of the toxin to the spinal cord with effects on contralateral and distant muscles, contributing to a “release” of the movements of the dominant hand [22].

### 4.3 Bimanual voluntary activity

While sitting, both before and after injections, children who got BoNT-A in the arm moved both hands simultaneously more, timewise, compared to children treated in the legs only. This was an unexpected finding, as it was known that these children rarely used their most affected hand voluntarily. Again, a plausible explanation is that there were involuntary mirror movements in the more affected arm.

After BoNT-A treatment of the arm, the difference between the arms regarding acceleration increased, possibly indicating a reduction in the intensity of the mirror movements of the non-dominant arm when the dominant hand was used. Thus, bothersome mirror movements could be an indication for BoNT-A treatment, as other researchers have suggested [35]. Another possible explanation could be a release of the voluntary use of the dominant arm due to a retrograde BoNT-A spinal effect [22].

In children who only got injections in the legs, three weeks post injections, the bimanual time during voluntary activity doubled and at the same time, the children walked less. This can be interpreted that weakness in the legs promoted play activities while sitting down.

Although, the primary goals for the BoNT-A treatment were that the children would walk more and use the most affected hand more. However, when assessing movements in daily life, it could not be verified that these goals were met, possibly due to weakness after the injections [23, 24]. Despite this, many children and their caretakers ask for new injections roughly every 6 months, perhaps due to a feeling of becoming more at ease as a result of direct spasticity reduction and diminished co-contractions and mirror movements through alternative pharmacological pathways [36].

### 4.4 Limitations

This study is limited by the small number of patients, heterogeneity regarding subtype of CP, age, and muscles injected. However, the diversity reflects the variety of patients in daily practice. There were missing data as some patients did not participate in the entire follow-up. Some accelerometers were not sufficiently fixed to the body with the Velcro straps. An additional limitation is that no typically developing children were included for comparison on, for example, time spent ambulating. For arm use in daily life, there is no gold standard for comparison. Although uniaxial accelerometers show little disadvantage compared to the later developed triaxial accelerometer examining physical activity in preschool children or adolescents, future studies can use multiaxial accelerometers placed on all four limbs and around the waist on both children with CP and typically developing children, and use machine learning in the analyses process [37–39].

## 5 Conclusion

At least for a 3-week follow-up, real-world monitoring of children with CP with a time-matched four-accelerometer set-up is feasible and may provide novel and valuable information not obtained by standard clinical follow-up of BoNT-A injections. Three weeks after BoNT-A injections into the legs, the children walked less and the time spent walking had not recovered at the three-month follow-up. Children with more severe affliction of one hand moved that hand less than the other before the injections, and received BoNT-A in that arm to use it more. However, the observed effect was the opposite, so that the affected arm was moved less, and the better arm more. This type of accelerometer setup could be used in future botulinum toxin treatment studies in cerebral palsy, with the hypotheses that treatment sessions will decrease mirror movements and the time spent walking.

## Supplementary Material

Supplementary Material
