# Supplementary material for: Time-matched accelerometers on limbs and waist in children with CP give new insights into real-life activities after botulinum toxin treatment: A proof of concept study
Source: J Pediatr Rehabil Med. 2023 Apr 6;16(1):125–37. doi: 10.3233/PRM-210112 (PMC10116137; doi:10.3233/PRM-210112)
Supplement: Supplementary Material [file prm-16-prm210112-s001.docx]

Appendix

**Statistical methods:** The data were analysed by using a linear mixed model (LMM) with maximum likelihood estimation. LMM handles data where observations are not independent and correctly models correlated errors. The LMM, therefore, provides the flexibility to model not only the mean of a response variable, but its covariance structure as well. LMM is an extension of the general linear model, in which factors and covariates are assumed to have a linear relationship to the dependent variable to better support analysis of a continuous dependent for repeated measures: where observations are correlated rather than independent.  LMM uses maximum likelihood estimation to estimate these parameters and supports more variations and data options. It modeled individual change over time and examined the effects of covariates on group differences. Normal probability plots, residual plots and Cook’s distance were used to assess the model assumptions. Analysis of residuals was also used to detect outliers visually. Cook's distance was examined to identify influential cases. The level of significance was specified at 5%.

**Result:** No violations of the assumptions of linearity and equal variance were observed. No outliers and influential cases were detected. Mild deviations from normality were observed. But as LMM is a very strong statistical method the results were accepted as valid.

**References**

Garson, G. D. (Ed.) (2013). Hierarchical linear modeling: Guide and applications. SAGE Publications, Inc. <https://dx.doi.org/10.4135/9781483384450>

Hesselmann G. (2018). Applying Linear Mixed Effects Models (LMMs) in Within-Participant Designs With Subjective Trial-Based Assessments of Awareness-a Caveat. Frontiers in psychology, 9, 788. <https://doi.org/10.3389/fpsyg.2018.00788> 
